# Alcohol-Related and Negatively Valenced Cues Increase Motor and Oculomotor Disinhibition in Social Drinkers

**DOI:** 10.1037/pha0000011

**Published:** 2015-03-02

**Authors:** Andrew Jones, Matt Field

**Affiliations:** 1 Department of Psychological Sciences, University of Liverpool and UK Centre for Tobacco & Alcohol Studies, Liverpool, United Kingdom

**Keywords:** alcohol cues, antisaccade, arousal, disinhibition, stop-signal task

## Abstract

Our aim in the present study was to investigate the psychological mechanisms that underlie the disinhibiting effects of alcohol cues in social drinkers by contrasting motor and oculomotor inhibition after exposure to alcohol-related, emotional, and neutral pictures. We conducted 2 studies in which social drinkers completed modified stop-signal (laboratory) and antisaccade (online) tasks in which positive, negative, alcohol-related, and neutral pictures were embedded. We measured cue-specific disinhibition in each task, and investigated whether sex and drinking status moderated the effects of pictures on disinhibition. Across both studies, comparable increases in disinhibition were observed in response to both alcohol and negatively valenced pictures, relative to both positive and neutral pictures. These differences in disinhibition could not be explained by differences between picture sets in arousal or valence ratings. There was no clear evidence of moderation by sex or drinking status. Secondary analyses demonstrated that alcohol-specific disinhibition was not reliably associated with individual differences in alcohol consumption or craving. These results suggest that the disinhibiting properties of alcohol-related cues cannot be attributed solely to their valence or arousing properties, and that alcohol cues may have unique disinhibiting properties.

Disinhibition, the inability to stop, change, or delay an inappropriate response, is a fundamental feature of both executive functioning and impulsivity (Bickel, Jarmolowicz, Mueller, Gatchalian, & McClure, 2012). Disinhibition in motor and oculomotor domains has been studied using the stop-signal and antisaccade tasks, respectively, and poor performance on these tasks is associated with drug and alcohol-use disorders (Smith, Mattick, Jamadar, & Iredale, 2014). Deficits in motor and oculomotor disinhibition discriminate between alcoholics and healthy controls (Goudriaan, Oosterlaan, De Beurs, & Van Den Brink, 2006; Noël et al., 2013) and are predictive of drinking behavior in nondependent drinkers (Christiansen, Cole, Goudie, & Field, 2012; Roberts, Miller, Weafer, & Fillmore, 2014). Findings from recent prospective studies suggest that the relationship between motor disinhibition and alcohol use may be causal, because elevated disinhibition precedes changes in drinking behavior. For example, relatively slow or suboptimal development of motor inhibition increases the likelihood of alcohol involvement (Fernie et al., 2013), and problematic drinking (Nigg et al., 2006) in adolescents, and in adults disinhibition predicts the severity of dependence at follow-up (Rubio et al., 2008).

The (in)ability to effectively inhibit behavior is thought to function as a state, that is immediately responsive to “abrupt environmental, physiological, or emotional events” (De Wit, 2009). In a recent review, we highlighted situations that increase disinhibition and possibly lead to (re)uptake of substance use (Jones, Christiansen, Nederkoorn, Houben, & Field, 2013), such as the presence of drug-related cues. For example, when embedded into response-inhibition tasks, alcohol-related cues increase disinhibition compared with neutral cues in nondependent drinkers (Adams, Ataya, Attwood, & Munafò, 2013; Petit, Kornreich, Noël, Verbanck, & Campanella, 2012; Weafer & Fillmore, 2012, 2014), with alcohol-specific disinhibition distinguishing problem and nonproblem drinkers on a go/no-go task (Kreusch, Vilenne, & Quertemont, 2013). Furthermore, individual differences in the inhibitory response to alcohol cues are associated with hazardous drinking (Petit et al., 2012) and self-reported alcohol consumption (Weafer & Fillmore, 2014). Studies have also explored the effects of in vivo alcohol-cue exposure on disinhibition. Gauggel et al. (2010) asked dependent patients to sniff alcoholic drinks before completing a stop-signal task and found increases in stop-signal reaction time (SSRT, indicative of increased disinhibition) compared with sniffing water. Muraven and Shmueli (2006) obtained similar results with social drinkers. However, there are also some inconsistencies in the literature regarding the effects of alcohol cues on disinhibition. Attempts to replicate effects obtained with the in vivo “drink-sniffing” paradigm (Muraven & Shmueli, 2006; Gauggel et al., 2010) have failed in both dependent (Mainz et al., 2012) and nondependent populations (Jones, Rose, Cole, & Field, 2013). In a study in which different cues were embedded in a stop-signal task, Nederkoorn, Baltus, Guerrieri, and Wiers (2009) reported no difference in disinhibition produced by alcohol-related, soft-drink, mildly erotic, or neutral cues, although they reported an overall impairment in disinhibition in heavy-drinking women.

The psychological mechanisms that underlie alcohol-cue-induced disinhibition have yet to be fully investigated, but at least three hypotheses have been identified. We have labeled these hypotheses based on our interpretation of predictions made by different theorists. According to the *emotional congruency hypothesis* (Guitart-Masip, Duzel, Dolan, & Dayan, 2014; Harlé, Shenoy, & Paulus, 2013; McLaren & Verbruggen, in press), negatively valenced cues trigger behavioral avoidance and inhibitory processes whereas positively valenced cues trigger behavioral approach and the suppression of inhibitory processes. Given that social drinkers perceive alcohol cues as positively valenced (Herrmann, Weijers, Wiesbeck, Boning, Fallgatter, 2001), their valence may account for their disinhibiting properties and there should be a clear relationship between the valence of different cues and their effects on disinhibition. A competing hypothesis (*arousal competition hypothesis*) suggests that arousing stimuli should lead to increased disinhibition through competition for executive resources. According to this account, the capture of attention by arousing stimuli draws resources away from the preparation of the inhibitory response (de Houwer & Tibboel, 2010; Pessoa, Padmala, Kenzer, & Bauer, 2012). Given that social drinkers perceive alcohol cues as arousing (Carter & Tiffany, 1999), arousal evoked by the cues may account for their disinhibiting properties, and there should be a clear relationship between the arousing properties of different cues and their effects on disinhibition. A final hypothesis suggests that substance-related cues have unique disinhibiting properties because their presence depletes self-control resources via suppression of temptations (*unique disinhibition hypothesis*). According to this account, these effects cannot be attributed to positive valence or arousing features of the cues, (Muraven & Shmueli, 2006; Shmueli & Prochaska, 2009).

Experimental studies have provided little support for the emotional congruency hypothesis. Both Verbruggen and de Houwer (2007) and Rebetez, Rochat, Billieux, Gay, and Van der Linden (2014) demonstrated increased disinhibition in response to positive stimuli compared with neutral stimuli, however they also observed increases in disinhibition in response to negative stimuli (see also Kalanthroff, Cohen, and Henik (2013)). In support of the arousal competition hypothesis, a second study by Verbruggen and de Houwer (2007) demonstrated that the disinhibiting effects of both positively and negatively valenced cues could be attributed to the subjective arousal ratings for those cues, rather than their valence per se. This was further supported by de Houwer and Tibboel (2010) who reported similar findings when using a go/no-go task to measure disinhibition.

To our knowledge, no previous studies have investigated the psychological mechanisms that underlie disinhibition evoked by alcohol-related cues. To achieve this aim, in the present studies we compared the effects of alcohol-related and positive, negative and neutral cues on motor and oculomotor disinhibition. In Experiment 1, we implemented a similar design to Nederkoorn et al. (2009) by embedding images into the stop-signal task. In Experiment 2, we applied the same image sets to an antisaccade task adapted from Noël et al. (2013), because the effects of concurrent exposure to alcohol cues are yet to be investigated in the domain of oculomotor inhibitory control. We used four picture sets; positive, negative, alcohol-related, and neutral, which were independently rated on continua of valence and arousal. These picture ratings revealed that negative cues were rated as most arousing, followed by positive, alcohol, and neutral cues. Alcohol cues were also rated as relatively neutral in terms of their valence.

We investigated three competing accounts of the effects of alcohol cues on disinhibition. Based on ratings of valence and arousal given to our pictures, the emotional congruency hypothesis would predict disinhibition in response to positive pictures relative to alcohol-related and neutral pictures (which should not differ from each other), but improved inhibitory control in response to negative pictures. Competing predictions are made by the arousal competition hypothesis: Disinhibition should be most pronounced in response to negative pictures, followed by positive pictures, alcohol pictures, and finally neutral pictures. Finally, the unique disinhibition hypothesis posits that alcohol-related cues should evoke the largest increases in disinhibition, even though they sit intermediate to the other pictures in terms of arousal and valence ratings. In accordance with previous research, we also examined whether disinhibition was modulated by drinking status and sex (Nederkoorn et al., 2009; Scaife & Duka, 2009). Finally, we examined whether disinhibition in response to alcohol cues would be associated with individual differences in alcohol use and problem-drinking measures, as has been reported previously (Petit et al., 2012).

## Experiment 1

### Method

#### Participants

Sixty-four social drinkers (32 male) were recruited from the University of Liverpool and wider community using advertisements on campus and via the Internet. To take part in the study, participants had to self-report consuming alcohol on at least one occasion per week. Exclusion criteria were self-reported history of, or treatment for, alcohol dependence or attention deficit hyperactivity disorder (ADHD). We applied these exclusion criteria because repeated presentation of alcohol cues to participants with alcohol-use disorders would have been unethical, and because ADHD is reliably associated with poor inhibitory control (Groman, James, & Jentsch, 2009) and could therefore have distorted our results. The study was approved by the University of Liverpool Research Ethics Committee, and all participants provided informed consent before taking part.

#### Questionnaires

Participants provided basic demographic information before completing a short questionnaire battery. The battery comprised the 2-week timeline follow-back (TLFB; Sobell & Sobell, 1992); a retrospective diary of their alcohol use over the previous fortnight (alcohol use calculated in UK units, 1 UK unit = 8 g of alcohol); the Alcohol Use Disorders Identification Test (AUDIT; Babor, Higgins-Biddle, Saunders, & Monteiro, 2001), which is a measure of hazardous drinking; the “right now” version of the Approach and Avoidance of Alcohol Questionnaire (AAAQ; McEvoy, Stritzke, French, Lang, & Ketterman, 2004) as a measure of self-reported craving; and the Barratt Impulsiveness Scale, Version 11 (BISv11; Patton, Stanford, & Barratt, 1995), assessing trait impulsiveness.

#### Pictorial stimuli

Four sets of 10 images were used; positive, negative, alcohol-related, and neutral. All images were 110 × 145 mm. Positive, negative and neutral images were taken from the *International Affective Picture System* (*IAPS*; Lang, Bradley, & Cuthbert, 1997) and initially chosen based on normative ratings of pleasantness in the technical report. Pictures containing images of food or drink were avoided (for detailed information, see Table 1). Alcohol images were taken from our previous research (Jones et al., 2012). To obtain valence and arousal ratings for the pictures, we recruited a unique sample of 20 participants and asked them to rate each of the images (see Table 2). Valence and arousal ratings were not obtained from the participants who completed the main experiments because we were concerned that repeated exposure to the images may have led to habituation of the response to them.[Table-anchor tbl1][Table-anchor tbl2]

#### Stop-signal task

Motor inhibition was measured using a modified stop-signal task (Logan, Schachar, & Tannock, 1997). The standard task requires participants to rapidly categorise arbitrary stimuli using a button press, these are “go trials.” On a minority of trials, this categorization response is interrupted by an auditory stimulus (the “stop signal”) and participants are required to inhibit their categorization response if they hear this signal. A modified version of the task was used that required a response to images. These images were rotated by 5 degrees clockwise or counterclockwise and participants were required to distinguish the rotation by pressing a designated key on the keyboard. We used a fixed delay version of the task with stop-signal delays of 100, 200, 300, & 400 ms (Logan, Cowan, & Davis, 1984). Delays were presented in random order and equally across each picture type. Participants completed eight practice trials with feedback on their responses before completing three test blocks of 128 trials. Each test block consisted of 96 go trials (24 for each picture type) and 32 stop trials (eight for each picture type, two at each stop-signal delay). The task was programmed using Inquisit 2.0 (Millisecond Software, Seattle, WA) and presented using a standard laptop.

SSRT was calculated using the integration method (Verbruggen & Logan, 2009). This involves rank ordering reaction times (RTs) on go trials. The *n*th RT is then selected based on the probability of responding at a given stop-signal delay. The stop-signal delay was subtracted from the *n*th RT and averaged over the delays for each picture type.

#### Procedure

Participants attended the laboratory between midday and 6 p.m. and provided informed consent before providing a breath alcohol sample (all participants had a breath alcohol level of 0). They then completed the modified stop-signal task, which took approximately 15 min. Following completion of the task, they completed the demographic and alcohol questionnaires. Participants were then debriefed, thanked, and offered course credit or a £5 shopping voucher as compensation for their time.

### Results

#### Demographics

Mean age of the participants was 22.34 (±3.51) years, they drank an average of 21.61 (± 14.84) units of alcohol per week, and had AUDIT scores of 12.61 (±6.04). Men drank significantly more than women: 25.96 ± 17.32 compared with 17.25 ± 10.54 units: *t*(62) = 2.43, *p* < .05. Descriptive statistics for the BIS (Patton et al., 1995) and AAAQ (McEvoy et al., 2004) are available on request. For subsequent analyses, a median split on fortnightly alcohol consumption, separately for men and women, was performed to create a heavy versus light drinking group, as in Nederkoorn et al. (2009).

#### Go reaction times

**Figure 1 fig1:**
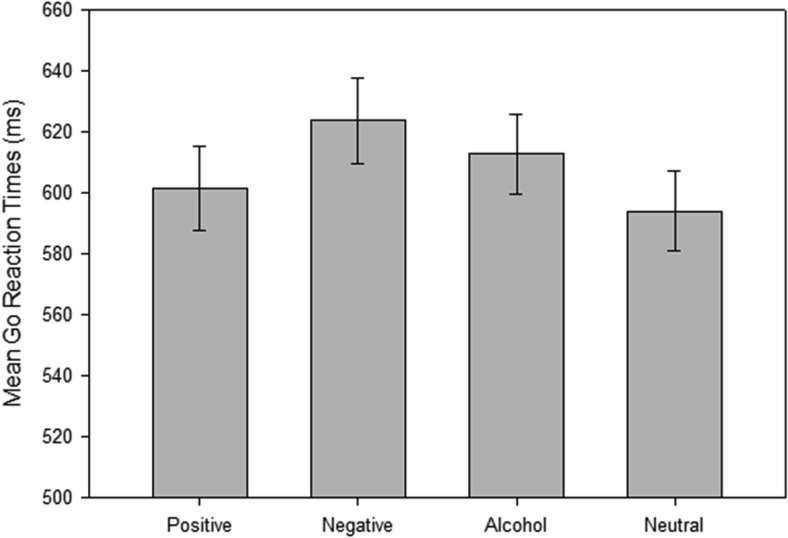
Mean Go Reaction Times scores (and standard errors) for each picture set during the stop-signal task.

Outliers and extreme data were removed using a trimming procedure similar to previous research (Verbruggen & De Houwer, 2007): RTs faster than 200 ms, or more than three standard deviations above the individual mean, were removed prior to analysis. Go Reaction times were analyzed using a mixed 4 (picture type: positive vs. negative vs. alcohol vs. neutral) × 2 (sex: male vs. female) × 2 (drinking status: heavy vs. light) analysis of variance (ANOVA; see Figure 1). There was a significant main effect of picture type on RTs, *F*(3, 180) = 15.70, *p* < .01, η_p_^2^ = .207. RTs were slowest to negative cues, followed by alcohol cues, then positive, then neutral cues (*t*s > 2.31, *p*s < .05). There were no significant interactions with sex or drinking status (*F*s < 1.25, *p*s > .10).[Fig-anchor fig1]

#### Inhibition errors

The mean inhibition error rate collapsed across picture type was 27.16 (*SD* = 12.90), which means that participants failed to inhibit on 26.07% of stop-signal trials, on average. Inhibition errors were analyzed using a mixed 4 × (picture type: positive vs. negative vs. alcohol vs. neutral) 2 × (sex: male vs. female) × 2 (drinking status: heavy vs. light) ANOVA. There was no significant main effect of picture type, *F*(1, 189) = 1.31, *p* > .10, η_p_^2^ = .021 and no significant interactions with sex or drinking status, *F*s< 0.90, *p*s > .10.

#### SSRT

**Figure 2 fig2:**
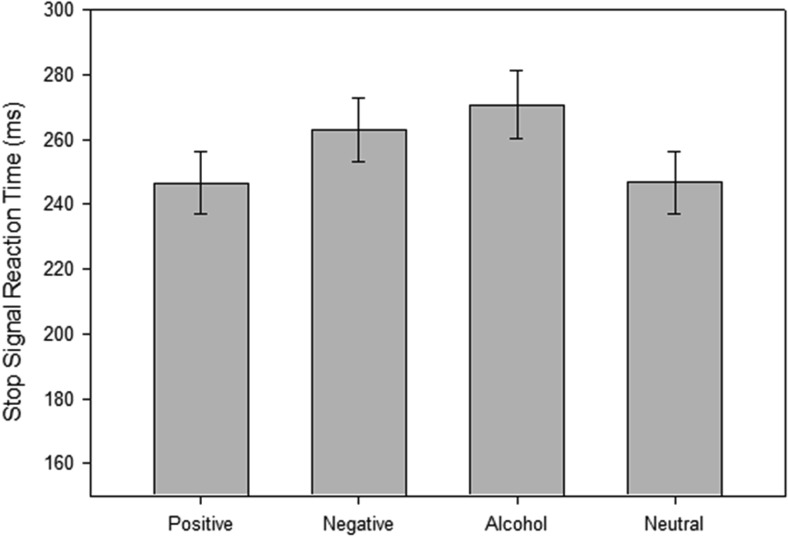
Mean Stop Signal Reaction Time (SSRT) scores (and standard errors) for each picture set during the stop-signal task.

SSRTs (see Figure 2) were analyzed using a mixed 4 × (picture type: positive vs. negative vs. alcohol vs. neutral) 2 × (sex: male vs. female) × 2 (drinking status: heavy vs. light) ANOVA. There was a significant main effect of picture type, *F*(1, 189) = 2.87, *p* < .05, η_p_^2^ = .046. Planned comparisons revealed that SSRT was significantly longer for alcohol cues than for neutral cues, *t*(63) = 2.24, *p* < .05, *d* = 0.28 and positive cues, *t*(63) = 2.36, *p* < .05, *d* = 0.29. SSRT was also significantly longer for negative compared with neutral cues, *t*(63) = 1.71, *p* < .05, *d* = 0.21. There was no difference between alcohol and negative cues, *t*(63) = 0.80, *p* > .10. There were no significant interactions with sex or drinking status (*F*s < 1.01, *p*s > .10).[Fig-anchor fig2]

#### Correlations

We examined whether individual differences in drinking variables: alcohol consumption, AUDIT (Babor et al., 2001) or AAAQ (McEvoy et al., 2004) subscales were associated with (a) alcohol-specific disinhibition, which was calculated by subtracting SSRT during neutral images from SSRT during alcohol images, or (b) overall disinhibition, which was computed as the mean SSRT across all picture types. There was a significant positive correlation between AAAQ-obsessed and alcohol-specific disinhibition, *r* = .27, *p* < .05 however this did not remain significant after correcting for multiple comparisons. All other correlations were not statistically significant (*r*s < −.20, *p*s > .10).

### Discussion

Both alcohol-related and negatively valenced pictures increased motor disinhibition relative to neutral pictures. We also observed a small positive correlation between the disinhibiting effects of alcohol cues and alcohol craving. These findings suggest that the disinhibiting properties of alcohol cues cannot simply be attributed to their valence or arousing properties, and they are consistent with the unique disinhibition rather than the emotional congruency or arousal competition accounts.

## Experiment 2

### Method

#### Participants

Participants (*N* = 117; 45 male) were recruited via the Internet to this web-based study, as well as through advertisements on the university intranet, social media, and a crowd-sourcing website (Crowd Flower). Inclusion and exclusion criteria were the same as the first experiment, however, participants also had to have access to the Internet using a Windows operating system and a keyboard. The study was approved by the University of Liverpool Research Ethics Committee. Participants took part for course credit, the chance to enter a prize draw, or a small financial reward on the crowd-source website.

#### Questionnaires

Participants completed the AUDIT (Babor et al., 2001) and a 1-week TLFB. They also completed a simplified measure of alcohol craving “Please rate your craving for alcohol on a scale of 0 (*no craving*) to 100 (*extreme craving*)”. Finally, participants were asked to state when they had last consumed an alcoholic drink from five possible options: less than 2 hr ago (0.85%), earlier today (2.56%), yesterday (24.78%), a few days ago (47.86%), or last week (23.93%). We excluded participants from subsequent analyses if they self-reported consuming alcohol on the day of the test (*n* = 4) to ensure that inhibitory control was not influenced by acute alcohol effects (Rose & Grunsell, 2008).

#### Antisaccade task

Oculomotor inhibition was measured using a modified antisaccade task based on one reported by Noël et al. (2013). The same picture sets from Experiment 1 were used. On each trial of the task, participants were presented with a fixation cross for 500 ms before a picture (positive, negative, neutral, or alcohol-related) appeared on either the left or right side of the screen for 225 ms. This image then disappeared and on the opposite side of the screen a small target stimulus, a white arrow pointing up, down or left was presented for 150 ms before being masked by a gray square. Participants were told to indicate the direction in which the arrow was pointing with a key press response. The short presentation time of the target stimulus requires participants to inhibit looking at the initial picture presentation to increase their chances of a correct response.

There was a practice block of 12 trials before five blocks of 24 trials. Picture types were counterbalanced across presentation (left, right) and presented in a random order on each block. Target stimulus orientation (up, down, left) was also presented randomly, but with equal probability. The outcome measure was the proportion of correct responses across picture type. The task was programmed in Inquisit 3.0 and hosted via Inquisit Web (Millisecond Software, Seattle, WA).

#### Procedure

Participants clicked a link that sent them to the secure site hosting the experiment. They were shown an information screen before being asked to provide informed consent. Participants were then given instructions for the antisaccade task before they completed it, which took approximately 6 min. Upon completion of the task participants were given the AUDIT (Babor et al., 2001), one week TLFB, and questions about craving and recent alcohol consumption before receiving an online debriefing.

### Results

Mean age of the participants was 24.78 (±7.72) years, they drank on average of 20.06 (±18.00) units per week and had AUDIT scores of 10.63 (±6.34). There were no significant sex differences in units consumed (men = 21.94 ±16.55, women = 18.99 ± 18.80; *t*_112_ = 0.84, *p* > .10), AUDIT scores (men = 9.51 ± 5.22, women = 11.26 ± 6.85; *t*_112_ = 1.42, *p* > .10), or craving (men = 26.49 ± 25.91, women = 18.39 ± 24.27; *t*_112_ = 1.66, *p* > .05).

#### Inhibition errors

**Figure 3 fig3:**
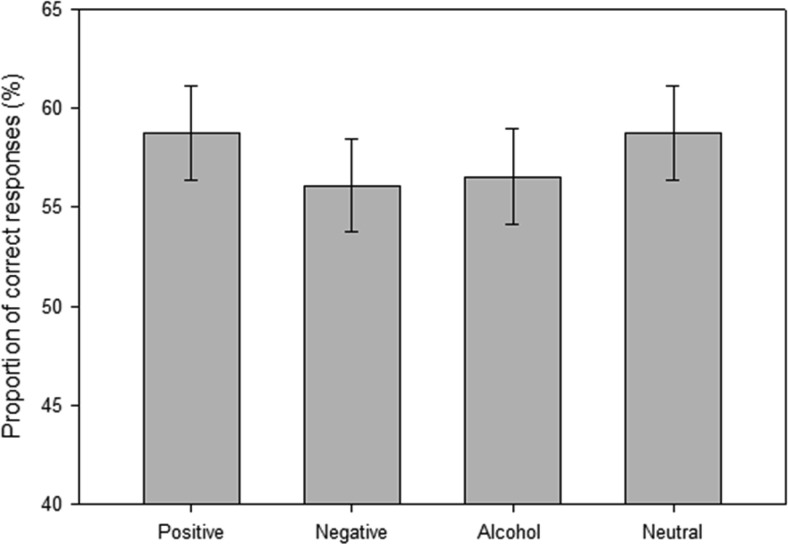
Mean proportion of correct responses (and standard errors) for each picture set during the anti-saccade task.

Proportion of correct responses to the target stimuli were analyzed using a mixed 4 (picture type: positive vs. negative vs. alcohol vs. neutral) × 2 (sex: male vs. female) × 2 (drinking status: heavy vs. light) ANOVA (see Figure 3). There was a significant main effect of picture type, *F*(3, 327) = 3.64, *p* < .05, η_p_^2^ = .032. Planned comparisons demonstrated that alcohol cues led to more errors than both positive, *t*(112) = 2.30, *p* < .05, *d* = 0.21, and neutral, *t*(112) = 2.24, *p* < .05, *d* = 0.21 cues. Negative cues also led to more errors than positive, *t*(112) = 2.94, *p* < .01, *d* = 0.28 and neutral cues, *t*(112) = 2.70, *p* < .01, *d* = 0.26. There was no significant difference between negative and alcohol cues, *t*(112) = 0.47, *p* > .10. There was also a significant Picture Type × Drinking Status interaction, *F*(3, 327) = 2.99, *p* < .05, η_p_^2^ = .027. Running the repeated-measures ANOVA separately demonstrated a significant effect of picture type in light drinkers, *F*(3, 168) = 5.99, *p* < .01, η_p_^2^ = .097, with no significant effect of picture type in heavy drinkers, *F*(3, 165) = 2.26, *p* = .08, η_p_^2^ = .039. Paired-samples *t* tests demonstrated that light drinkers had more errors following negative, *t*(56) = 3.94, *p* < .001, alcohol, *t*(56) = 4.23, *p* < .001, and neutral cues, *t*(112) = 2.13, *p* < .05 than with positive cues. There were no between-groups differences between heavy and light drinkers in proportion of errors on any picture type (*t*s < 1.00, *p*s >.10). There were no significant interactions involving sex, Picture Type × Sex Interaction, *F*(3, 327) = 1.32, *p* > .10; Picture Type × Drinking Status × Sex Interaction, *F*(3, 327) = 0.52, *p* > .10.[Fig-anchor fig3]

#### Correlations

In line with Experiment 1, we examined whether individual differences in drinking variables (i.e., weekly alcohol consumption, AUDIT, or craving) were associated with alcohol-specific inhibition (i.e., proportion of errors on alcohol trials minus proportion of errors on neutral trials) or overall inhibition. However, there were no significant correlations (*r*s < .01, *p*s > .10).

### Discussion

The results from these two studies have demonstrated that alcohol-related and negatively valenced, highly arousing cues produce comparable increases in motor and oculomotor disinhibition in social drinkers. Although our results were consistent across two studies, they did not clearly support either the emotional congruency or the arousal competition hypotheses. The emotional congruency hypothesis predicts increased disinhibition in response to positively valenced cues but improved inhibition in response to negatively valenced cues: our studies actually demonstrated comparable increases in disinhibition in response to both negative and alcohol-related pictures, relative to both positive and neutral pictures (that did not differ from each other). The arousal competition hypothesis predicts increased disinhibition in response to the most arousing pictures. Although the elevated disinhibition in response to negative cues was consistent with this account, the similar increase in disinhibition in response to alcohol pictures, despite those pictures being rated as less arousing than both positive and negative pictures,was clearly inconsistent with this hypothesis.

To interpret the disinhibition findings, it is important to consider the effect of the different pictures on RTs on go trials. The pictures that evoked the largest increase in disinhibition (based on SSRT; Figure 2) also led to the slowing of RTs on go trials (see Figure 2), although the correspondence between these two measures was not perfect (RTs were slower to negative than alcohol-related cues, but these cues did not differ in SSRT). Negative stimuli led to a general “freezing” of motor activity, which led to a slowing of RT (de Houwer & Tibboel, 2010; Estes & Verges, 2008). This is generally supported by similar findings reported by Verbruggen and de Houwer (2007) and also Sagaspe et al. (2011), who demonstrated slowing of RTs to negative cues. However, previous studies that implemented the stop-signal task did not report RT data. To fully understand motivational modulation of inhibitory control, it is important to report RTs alongside measures of inhibitory control (Herrera, Speranza, Hampshire, & Bekinschtein, 2014).

The slowing of RTs cannot fully explain alcohol-specific disinhibition because negative cues led to greater slowing than alcohol cues. Overall, our findings suggest that alcohol cues have unique disinhibiting properties that cannot be attributed to their valence or arousing properties. Based on previous research, we speculate that suppression of momentary craving evoked by alcohol-related cues may have prompted a transient spike in disinhibition (Jones, Rose, et al., 2013; Muraven & Shmueli, 2006). In support of this, we found a weak positive correlation between alcohol-specific disinhibition and craving in Experiment 1, which supported findings from our previous research (Jones, Rose, et al., 2013); however, we did not replicate this finding in Experiment 2. Future research should focus on elucidating the exact mechanisms that underlie drug-specific inhibition. For example, an “attentional bias” toward alcohol-related cues (Field & Cox, 2008; Field, Mogg, Zetteler, & Bradley, 2004) might create competition for executive resources, and therefore indirectly contribute to their disinhibiting properties (Weafer & Fillmore, 2012).

In both studies, we found no significant relationships between individual differences in alcohol consumption or hazardous drinking and the effects of alcohol cues on disinhibition. Our findings contrast with those from other studies, which did demonstrate such associations (Kreusch et al., 2013; Petit et al., 2012). It appears that the disinhibiting properties of alcohol-related cues are not robustly associated with alcohol consumption or problem drinking (see also Adams et al., 2013). The inconsistency in the literature might be partly attributable to the wide variation in methods used (Smith, Mattick, Jamadar, Iredale, 2014; Stevens et al., 2014). Nonetheless, this inconsistency is problematic for theoretical models that posit that the disinhibiting effects of alcohol cues might contribute to excessive alcohol consumption and the development of alcohol-use disorders (Goldstein & Volkow, 2002; Jones, Christiansen, et al., 2013; Wiers et al., 2007).

There are a few limitations to these studies. Our picture sets were not balanced in terms of ratings of arousal and valence, however, this allowed for the opportunity to disentangle the two. The difficulty in matching picture sets on both valence and arousal has been highlighted previously (de Houwer & Tibboel, 2010; Verbruggen & De Houwer, 2007). Our ratings were also taken from different participants than those who completed the main studies, which means that we are unable to correlate individual differences in picture ratings with the effects of those pictures on inhibitory control. Our decision to record picture ratings in a different sample from the participants who completed the inhibitory control tasks was motivated by concerns that participants’ emotional response to the pictures might have habituated as a consequence of repeated exposure. Furthermore, other studies have demonstrated that if participants make inhibitory responses to positive and appetitive stimuli, these stimuli become devalued (Veling, Holland, & van Knippenberg, 2008). Therefore, picture ratings would be difficult to interpret even if administered after the inhibitory control task. Nevertheless, future researchers should attempt to collect picture ratings from the same individuals who completed inhibitory control tasks TO improve our understanding of underlying processes. Second, data collection via the Internet can lack experimental control; therefore we should be cautious until the results from the second study are replicated in laboratory settings. However, our error rates were similar to those reported in an earlier study that administered the same task in a laboratory (Noël et al., 2013), and other cognitive tasks tend to yield comparable performance indices, whether administered online or in the laboratory (Crump, McDonnell, & Gureckis, 2013; Houben & Wiers, 2008).

To conclude, we presented two studies demonstrating that both motor and oculomotor disinhibition can be temporarily increased by alcohol-related and negatively valenced images. These findings suggest that alcohol-related cues have unique disinhibiting properties which cannot be explained as a result of the valence or arousal attributed to these cues.

## Figures and Tables

**Table 1 tbl1:** Details of Images Taken From the International Affective Picture System (IAPS)

Positive		Negative		Neutral	
IAPS code	Description	IAPS code	Description	IAPS code	Description
1999	Mickey	1280	Rat	2880	Shadow
1603	Butterfly	1300	Pit bull	5535	Still life
1463	Kittens	6550	Attack	5900	Desert
5480	Fireworks	6570	Suicide	6150	Outlet
5626	Hangglider	2691	Riot	7025	Stool
8162	Hot air ballon	8231	Boxer	7140	Bus
8190	Skier	9040	Starving child	7150	Umbrella
8461	Happy teens	9921	Fire	7175	Lamp
1601	Giraffes	9594	Injection	7491	Building
2040	Baby	9410	Soldier	7545	Ocean
*Note.* Codes and descriptions taken from the *IAPS Technical Report*.					

**Table 2 tbl2:** Mean Scores (±SDs) for Valence and Arousal for the Positive, Negative, Alcohol, and Neutral Picture Sets

Variable	Positive	Negative	Alcohol	Neutral	*F* value	*p*
Valence	7.10 (0.79)	2.27 (0.96)	5.08 (1.15)	4.77 (0.74)	85.09	<.001
Arousal	3.59 (1.49)	6.20 (1.67)	2.50 (1.50)	1.56 (0.45)	53.03	<.001
*Note.* Valence was rated on a 1–9 scale with the following anchors: 1 = *negative*, 5 = *neutral*, 9 = *positive*). Arousal was rated on a 1–9 scale with the following anchors; 1 = *not at all*, 9 = *extremely*. Arousal: all ratings significantly different from others (*t*s > 2.85, *p*s < .05). Valence: all ratings significantly different (*t*s > 6.52, *p*s < .001) aside from no significant difference between alcohol and neutral picture sets, *t*(19) = 1.20, *p* > .10.						

## References

[c1] Adams S., Ataya A. F., Attwood A. S., & Munafò M. R. (2013). Effects of alcohol on disinhibition towards alcohol-related cues. Drug and Alcohol Dependence, 127, 137–142 10.1016/j.drugalcdep.2012.06.025 22841455

[c2] Babor T. F., Higgins-Biddle J. C., Saunders J. B., & Monteiro M. G. (2001). The Alcohol Use Disorders Identification Test: Guidelines for use in primary care. Geneva, Switzerland: World Health Organisation, Department of Mental Health and Substance Dependence.

[c3] Bickel W. K., Jarmolowicz D. P., Mueller E. T., Gatchalian K. M., & McClure S. M. (2012). Are executive function and impulsivity antipodes? A conceptual reconstruction with special reference to addiction. Psychopharmacology, 221, 361–387 10.1007/s00213-012-2689-x 22441659 PMC4035182

[c4] Carter B. L., & Tiffany S. T. (1999). Meta-analysis of cue-reactivity in addiction research. Addiction, 94, 327–340 10.1046/j.1360-0443.1999.9433273.x 10605857

[c5] Christiansen P., Cole J. C., Goudie A. J., & Field M. (2012). Components of behavioural impulsivity and automatic cue approach predict unique variance in hazardous drinking. Psychopharmacology, 219, 501–510 10.1007/s00213-011-2396-z 21735071

[c6] Crump M. J. C., McDonnell J. V., & Gureckis T. M. (2013). Evaluating Amazon’s Mechanical Turk as a tool for experimental behavioral research. PLoS ONE, 8, 10.1371/journal.pone.0057410 PMC3596391 23516406

[c7] De Houwer J., & Tibboel H. (2010). Stop what you are not doing! Emotional pictures interfere with the task not to respond. Psychonomic Bulletin & Review, 17, 699–703 10.3758/PBR.17.5.699 21037169

[c8] de Wit H. (2009). Impulsivity as a determinant and consequence of drug use: A review of underlying processes. Addiction Biology, 14, 22–31 10.1111/j.1369-1600.2008.00129.x 18855805 PMC3640851

[c9] Estes Z., & Verges M. (2008). Freeze or flee? Negative stimuli elicit selective responding. Cognition, 108, 557–565 10.1016/j.cognition.2008.03.003 18433742

[c10] Fernie G., Peeters P., Gullo M. J., Christiansen P., Cole J., Sumnall H., & Field M. (2013). Multiple behavioural impulsivity tasks predict prospective alcohol involvement in adolescents. Addiction, 108, 1916–1923 10.1111/add.12283 23795646 PMC4230409

[c11] Field M., & Cox W. M. (2008). Attentional bias in addictive behaviors: A review of its development, causes, and consequences. Drug and Alcohol Dependence, 97, 1–20 10.1016/j.drugalcdep.2008.03.030 18479844

[c12] Field M., Mogg K., Zetteler J., & Bradley B. P. (2004). Attentional biases for alcohol cues in heavy and light social drinkers: The roles of initial orienting and maintained attention. Psychopharmacology, 176, 88–93 10.1007/s00213-004-1855-1 15071718

[c13] Gauggel S., Heusinger A., Forkmann T., Boecker M., Lindenmeyer J., Cox W. M., & Staedtgen M. (2010). Effects of alcohol cue exposure on response inhibition in detoxified alcohol-dependent patients. Alcoholism: Clinical and Experimental Research, 34, 1584–1589 10.1111/j.1530-0277.2010.01243.x 20586755

[c14] Goldstein R. Z., & Volkow N. D. (2002). Drug addiction and its underlying neurobiological basis: Neuroimaging evidence for the involvement of the frontal cortex. The American Journal of Psychiatry, 159, 1642–1652 10.1176/appi.ajp.159.10.1642 12359667 PMC1201373

[c15] Goudriaan A. E., Oosterlaan J., de Beurs E., & van den Brink W. (2006). Neurocognitive functions in pathological gambling: A comparison with alcohol dependence, Tourette syndrome and normal controls. Addiction, 101, 534–547 10.1111/j.1360-0443.2006.01380.x 16548933

[c16] Groman S. M., James A. S., & Jentsch J. D. (2009). Poor response inhibition: At the nexus between substance abuse and attention deficit/hyperactivity disorder. Neuroscience and Biobehavioral Reviews, 33, 690–698 10.1016/j.neubiorev.2008.08.008 18789354 PMC2728075

[c17] Guitart-Masip M., Duzel E., Dolan R., & Dayan P. (2014). Action versus valence in decision making. Trends in Cognitive Sciences, 18, 194–202 10.1016/j.tics.2014.01.003 24581556 PMC3989998

[c18] Harlé K. M., Shenoy P., & Paulus M. P. (2013). The influence of emotions on cognitive control: Feelings and beliefs-where do they meet? Frontiers in Human Neuroscience, 7, 508. 24065901 10.3389/fnhum.2013.00508 PMC3776943

[c19] Herrera P. M., Speranza M., Hampshire A., & Bekinschtein T. A. (2014). Monetary rewards modulate inhibitory control. Frontiers in Human Neuroscience, 8, 257. 24860469 10.3389/fnhum.2014.00257 PMC4026705

[c20] Herrmann M. J., Weijers H. G., Wiesbeck G. A., Böning J., & Fallgatter A. J. (2001). Alcohol cue-reactivity in heavy and light social drinkers as revealed by event-related potentials. Alcohol and Alcoholism, 36, 588–593 10.1093/alcalc/36.6.588 11704627

[c21] Houben K., & Wiers R. W. (2008). Measuring implicit alcohol associations via the Internet: Validation of Web-based implicit association tests. Behavior Research Methods, 40, 1134–1143 10.3758/BRM.40.4.1134 19001405

[c22] Jones A., Christiansen P., Nederkoorn C., Houben K., & Field M. (2013). Fluctuating disinhibition: Implications for the understanding and treatment of alcohol and other substance use disorders. Frontiers in Psychiatry, 4, 140. 24155728 10.3389/fpsyt.2013.00140 PMC3804868

[c23] Jones A., Hogarth L., Christiansen P., Rose A. K., Martinovic J., & Field M. (2012). Reward expectancy promotes generalized increases in attentional bias for rewarding stimuli. The Quarterly Journal of Experimental Psychology, 65, 2333–2342 10.1080/17470218.2012.686513 22631033

[c24] Jones A., Rose A. K., Cole J., & Field M. (2013). Effects of alcohol cues on craving and ad libitum alcohol consumption in social drinkers: The role of disinhibition. Journal of Experimental Psychopathology, 4, 239–249 10.5127/jep.031912

[c25] Kalanthroff E., Cohen N., & Henik A. (2013). Stop feeling: Inhibition of emotional interference following stop-signal trials. Frontiers in Human Neuroscience, 7, 78. 23503817 10.3389/fnhum.2013.00078 PMC3596782

[c26] Kreusch F., Vilenne A., & Quertemont E. (2013). Response inhibition toward alcohol-related cues using an alcohol go/no-go task in problem and non-problem drinkers. Addictive Behaviors, 38, 2520–2528 10.1016/j.addbeh.2013.04.007 23773960

[c27] Lang P. J., Bradley M. M., & Cuthbert B. N. (1997). International Affective Picture System (IAPS): Technical manual and affective ratings. Gainesville, FL: University of Florida.

[c28] Logan G. D., Cowan W. B., & Davis K. A. (1984). On the ability to inhibit simple and choice reaction time responses: A model and a method. Journal of Experimental Psychology: Human Perception and Performance, 10, 276–291 10.1037/0096-1523.10.2.276 6232345

[c29] Logan G. D., Schachar R. J., & Tannock R. (1997). Impulsivity and inhibitory control. Psychological Science, 8, 60–64 10.1111/j.1467-9280.1997.tb00545.x

[c30] Mainz V., Drüke B., Boecker M., Kessel R., Gauggel S., & Forkmann T. (2012). Influence of cue exposure on inhibitory control and brain activation in patients with alcohol dependence. Frontiers in Human Neuroscience, 6, 92. 22557953 10.3389/fnhum.2012.00092 PMC3340941

[c31] McEvoy P. M., Stritzke W. G. K., French D. J., Lang A. R., & Ketterman R. (2004). Comparison of three models of alcohol craving in young adults: A cross-validation. Addiction, 99, 482–497 10.1111/j.1360-0443.2004.00714.x 15049748

[c32] McLaren I. P. L., & Verbruggen F. (in press). Association and inhibition In Murphey R. A. & Honey R. C. (Eds.), The Wiley–Blackwell handbook on the cognitive neuroscience of learning. Hoboken, NJ: Wiley–Blackwell.

[c33] Muraven M., & Shmueli D. (2006). The self-control costs of fighting the temptation to drink. Psychology of Addictive Behaviors, 20, 154–160 10.1037/0893-164X.20.2.154 16784361

[c34] Nederkoorn C., Baltus M., Guerrieri R., & Wiers R. W. (2009). Heavy drinking is associated with deficient response inhibition in women but not in men. Pharmacology Biochemistry and Behavior, 93, 331–336 10.1016/j.pbb.2009.04.015 19409923

[c35] Nigg J. T., Wong M. M., Martel M. M., Jester J. M., Puttler L. I., Glass J. M., . . . Zucker R. A. (2006). Poor response inhibition as a predictor of problem drinking and illicit drug use in adolescents at risk for alcoholism and other substance use disorders. Journal of the American Academy of Child & Adolescent Psychiatry, 45, 468–475 10.1097/01.chi.0000199028.76452.a9 16601652

[c36] Noël X., Van der Linden M., Brevers D., Campanella S., Verbanck P., Hanak C., . . . Verbruggen F. (2013). Separating intentional inhibition of prepotent responses and resistance to proactive interference in alcohol-dependent individuals. Drug and Alcohol Dependence, 128, 200–205 10.1016/j.drugalcdep.2012.08.021 22980674

[c37] Patton J. H., Stanford M. S., & Barratt E. S. (1995). Factor structure of the Barratt Impulsiveness Scale. Journal of Clinical Psychology, 51, 768–774 10.1002/1097-4679(199511)51:6<768::AID-JCLP2270510607>3.0.CO;2-1 8778124

[c38] Pessoa L., Padmala S., Kenzer A., & Bauer A. (2012). Interactions between cognition and emotion during response inhibition. Emotion, 12, 192–197 10.1037/a0024109 21787074 PMC3208031

[c39] Petit G., Kornreich C., Noël X., Verbanck P., & Campanella S. (2012). Alcohol-related context modulates performance of social drinkers in a visual go/no-go task: A preliminary assessment of event-related potentials. PLoS ONE, 7, 10.1371/journal.pone.0037466 PMC3355129 22616012

[c40] Rebetez M. M. L., Rochat L., Billieux J., Gay P., & Van der Linden M. (2014). Do emotional stimuli interfere with two distinct components of inhibition? Cognition and Emotion, 2, 1–9 10.1080/02699931.2014.922054 24885111

[c41] Roberts W., Miller M. A., Weafer J., & Fillmore M. T. (2014). Heavy drinking and the role of inhibitory control of attention. Experimental and Clinical Psychopharmacology, 22, 133–140 10.1037/a0035317 24611837 PMC4082663

[c42] Rose A. K., & Grunsell L. (2008). The subjective, rather than the disinhibiting, effects of alcohol are related to binge drinking. Alcoholism: Clinical and Experimental Research, 32, 1096–1104 10.1111/j.1530-0277.2008.00672.x 18445111

[c43] Rubio G., Jiménez M., Rodríguez-Jiménez R., Martínez I., Avila C., Ferre F., . . . Palomo T. (2008). The role of behavioral impulsivity in the development of alcohol dependence: A 4-year follow-up study. Alcoholism: Clinical and Experimental Research, 32, 1681–1687 10.1111/j.1530-0277.2008.00746.x 18631324

[c44] Sagaspe P., Schwartz S., & Vuilleumier P. (2011). Fear and stop: A role for the amygdala in motor inhibition by emotional signals. NeuroImage, 55, 1825–1835 10.1016/j.neuroimage.2011.01.027 21272655

[c45] Scaife J. C., & Duka T. (2009). Behavioural measures of frontal lobe function in a population of young social drinkers with binge drinking pattern. Pharmacology Biochemistry and Behavior, 93, 354–362 10.1016/j.pbb.2009.05.015 19497334

[c46] Shmueli D., & Prochaska J. J. (2009). Resisting tempting foods and smoking behavior: Implications from a self-control theory perspective. Health Psychology, 28, 300–306 10.1037/a0013826 19450035 PMC2736876

[c47] Smith J. L., Mattick R. P., Jamadar S. D., & Iredale J. M. (2014). Deficits in behavioural inhibition in substance abuse and addiction: A meta-analysis. Drug and Alcohol Dependence, 145C, 1–33 10.1016/j.drugalcdep.2014.08.009 25195081

[c48] Sobell L. C., & Sobell M. B. (1992). Timeline follow-back: A technique for assisting self-reported alcohol consumption In Litten R. Z. & Allen J. P. (Eds.), Measuring alcohol consumption: Psychosocial and biochemical methods (pp. 41–72). Totowa, NJ: Humana Press 10.1007/978-1-4612-0357-5_3

[c49] Stevens L., Verdejo-García A., Goudriaan A. E., Roeyers H., Dom G., & Vanderplasschen W. (2014). Impulsivity as a vulnerability factor for poor addiction treatment outcomes: A review of neurocognitive findings among individuals with substance use disorders. Journal of Substance Abuse Treatment, 47, 58–72 10.1016/j.jsat.2014.01.008 24629886

[c50] Veling H., Holland R. W., & van Knippenberg A. (2008). When approach motivation and behavioral inhibition collide: Behavior regulation through stimulus devaluation. Journal of Experimental Social Psychology, 44, 1013–1019 10.1016/j.jesp.2008.03.004

[c51] Verbruggen F., & De Houwer J. (2007). Do emotional stimuli interfere with response inhibition? Evidence from the stop-signal paradigm. Cognition and Emotion, 21, 391–403 10.1080/02699930600625081

[c52] Verbruggen F., & Logan G. D. (2009). Models of response inhibition in the stop-signal and stop-change paradigms. Neuroscience and Biobehavioral Reviews, 33, 647–661 10.1016/j.neubiorev.2008.08.014 18822313 PMC2696813

[c53] Weafer J., & Fillmore M. T. (2012). Alcohol-related stimuli reduce inhibitory control of behavior in drinkers. Psychopharmacology, 222, 489–498 10.1007/s00213-012-2667-3 22358851 PMC4301262

[c54] Weafer J., & Fillmore M. T. (2014). (in press). Alcohol-related cues potentiate alcohol impairment of behavioral control in drinkers. Psychology of Addictive Behaviors. Advance online publication 10.1037/adb0000013 PMC4333136 25134023

[c55] Wiers R. W., Bartholow B. D., van den Wildenberg E., Thush C., Engels R. C. M. E., Sher K. J., . . . Stacy A. W. (2007). Automatic and controlled processes and the development of addictive behaviors in adolescents: A review and a model. Pharmacology Biochemistry and Behavior, 86, 263–283 10.1016/j.pbb.2006.09.021 17116324

